# Can enhancement and suppression concurrently guide attention? An assessment at the individual level

**DOI:** 10.12688/f1000research.77430.1

**Published:** 2022-02-24

**Authors:** Tomoya Kawashima, Kaoru Amano

**Affiliations:** 1 Graduate School of Human Sciences, Osaka University, 1-2 Yamadaoka, Suita City, Osaka, 565-0871, Japan; 2 Center for Information and Neural Networks (CiNet), Advanced ICT Research Institute, National Institute of Information and Communications Technology (NICT), 1-4 Yamadaoka, Suita City, Osaka, 565-0871, Japan; 3 Graduate School of Information Science and Technology, The University of Tokyo, 7-3-1 Hongo, Bunkyo-ku, Tokyo, 113-8656, Japan

**Keywords:** Attention, Enhancement, Suppression, Visual search

## Abstract

Background: Although people can pay attention to targets while ignoring distractors, previous research suggests that target enhancement and distractor suppression work separately and independently. Here, we sought to replicate previous findings and re-establish their independence. Methods: We employed an internet-based psychological experiment. We presented participants with a visual search task in which they searched for a specified shape with or without a singleton. We replicated the singleton-presence benefit in search performance, but this effect was limited to cases where the target color was fixed across all trials. In a randomly intermixed probe task (30% of all trials), the participants searched for a letter among colored probes; we used this task to assess how far attention was separately allocated toward the target or distractor dimensions. Results: We found a negative correlation between target enhancement and distractor suppression, indicating that the participants who paid closer attention to target features ignored distractor features less effectively and vice versa. Averaged data showed no benefit from target color or cost from distractor color, possibly because of the substantial differences in strategy across participants. Conclusions: These results suggest that target enhancement and distractor suppression guide attention in mutually dependent ways and that the relative contribution of these components depends on the participants’ search strategy.

## Introduction

Owing to limitations in attentional capacity, we must attend selectively to goal-related items and ignore ones that are unrelated to our goals. Directing our visual attention toward an object with a specific feature dimension can help improve the detection of task-relevant items. The possession of prior information regarding the properties of a target is known to expedite visual search by enhancing the attention paid to target stimuli ( Vickery, King, & Jiang, 2005; Wolfe, Horowitz, Kenner, Hyle, & Vasan, 2004). Another means of accelerating visual search is to guide attention away from task-irrelevant items ( Arita, Carlisle, & Woodman, 2012; Woodman & Luck, 2007). For example, Arita *et al.* (2012) found that presenting prior information on a color to be ignored sped up the perceiver’s visual search. The participants in their study performed a visual search after providing a distractor color as a negative cue; target detection was found to be faster than in neutral trials, and the authors argued that observers can use their prior knowledge of distractor features to guide visual attention.

This distractor suppression has been reportedly achieved through extensive practice or learning ( Cunningham & Egeth, 2016; Zehetleitner, Goschy, & Müller, 2012; Geng, Won, & Carlisle, 2019). Cunningham and Egeth (2016) asked participants to perform a visual search task with negative or neutral cues (such as the words “Ignore Red” or “Neutral”) and found that the reaction time (RT) to the target was increased by negative cues in the first block of 72 trials; however, this difference decreased in subsequent blocks. These results suggest that observers can learn to suppress specific to-be-ignored features through considerable practice.

Further studies have shown that even a salient distractor can be suppressed ( Chang & Egeth, 2019, 2021; Gaspelin, Leonard, & Luck, 2015, Gaspelin & Luck, 2018a). Gaspelin *et al.* (2015) embedded probe tasks into visual search tasks to examine the distractor suppression effect. In the visual search task (70% of all trials), the target was defined by a shape (e.g., a diamond), and a singleton distractor was presented for half of the search trials. In the probe task (30% of all trials), alphabet letters were briefly presented (100 ms) among the search shapes, and the participants were asked to report as many letters as they could recall. The authors found that the RTs in the search task were faster when the singleton was presented ( *singleton-presence benefit*). In the probe task, the recall accuracy for probes at the singleton location was lower, suggesting that the benefit was not due to the rapid disengagement from the singleton. From these results, Gaspelin *et al.* (2015) proposed that a physically salient item can be actively suppressed before attentional capture (signal-suppression hypothesis ( Sawaki & Luck, 2010, 2013)).

This distractor suppression can be explained by two mechanisms: secondary inhibition or active suppression ( Chelazzi, Marini, Pascucci, & Turatto, 2019; Gaspelin & Luck, 2018b; van Moorselaa & Slagter, 2020). In the first, ignoring task-unrelated stimuli is performed by focusing attention onto the target representation. Even where the distractor is salient, distractor interference can be diminished through a top-down attentional setting that focuses on target features (feature-search mode) relative to the set focusing on salient items (singleton-detection mode ( Bacon & Egeth, 1994; Leber & Egeth, 2006)). Thus, target enhancement can indirectly suppress distractor representation simply because the distractors are not attended to (i.e., the attention is directed away or secondary inhibition). The second involves direct distractor suppression. Several studies that incorporate electroencephalography (EEG) have observed the distractor-suppression related distractor positivity (Pd) component in response to salient distractors without the N2pc (N2-posterior-contralateral) component, which represents attentional selection ( Luck, 2012; Luck & Hillyard, 1994), suggesting that the distractor can be suppressed without attentional selection ( Gaspelin & Luck, 2018a, b; Sawaki & Luck, 2010; Burra & Kerzel, 2013).

Chang and Egeth (2019, 2021) sought to determine whether singleton-presence benefit in visual search could be explained by target enhancement, distractor suppression, or both. Instead of using a memory-based probe task, as Gaspelin *et al.* (2015) did, Chang and Egeth (2019) asked participants to detect a probe target (“A” or “B”) presented in a probe in a forced-choice manner and found 9 milliseconds (ms) singleton-presence benefit in visual search. Critically, the probe target appeared in the target or distractor feature that had been presented in search trials such that target enhancement and distractor suppression could be assessed separately. Moreover, these authors revealed that the RT was faster when the probe target was in the target color (34 ms target-color benefit) and slower when it was in the distractor color (43 ms distractor-color cost), arguing that target enhancement and distractor suppression guide attention in separate and independent ways ( *enhancement-plus-suppression*: Chang & Egeth, 2019). Thus, target enhancement and distractor suppression work in parallel ( Andersen & Müller, 2010).

Although Chang and Egeth (2019) concluded that “observers can concurrently maintain two different attentional control processes and use either one of them as the occasion demands” (p. 1731), this concurrent enhancement and suppression are cognitively demanding. Although such attentional templates can be created implicitly through successive practice, attention should be allocated at the beginning of the experimental session to a specific feature, particularly to the to-be-suppressed items ( Cunningham & Egeth, 2016). Furthermore, several studies have shown that visual attention can be guided by only a single item in working memory ( Olivers, Peters, Houtkamp, & Roelfsema, 2011; van Moorselaar, Theeuwes, & Olivers, 2014). van Moorselaar *et al.* (2014) required participants to conduct a visual search while holding a variable number of colors in working memory and found that attentional guidance through working memory representation was obtained only when a single color appeared. First, these results suggest that it is difficult to guide attention simultaneously by more than two representations, indicating a possible cognitive demand for maintaining representations for both enhancement and suppression (but see Bahle, Beck, & Hollingworth, 2018; Bahle, Thayer, Mordkoff, & Hollingworth, 2020). Second, attending to target information may be preferable to ignoring distractor features because enhancement and suppression do not have the same efficiency in attentional guidance. The effects of negative cues have been widely reported to be smaller than those for positive cues ( Arita *et al.*, 2012; Beck & Hollingworth, 2015; Becker, Hemsteger, & Peltier, 2016). Even within the same search settings, distractor inhibition mediated by negative cues has been shown to be inefficient relative to target enhancement mediated by positive cues ( Kawashima & Matsumoto, 2018). When both positive and negative cues are provided, participants selectively encode positive information for visual search ( Rajsic, Carlisle, & Woodman, 2020), although some studies suggest simultaneous guidance of target enhancement and distractor rejection ( Stiwell & Vecera, 2020; Beck, Luck, & Hollingworth, 2018). Based on the reported individual differences in selecting a search strategy ( Boot, Becic, & Kramer, 2009) and the tendency to avoid cognitive demands ( Kool, McGuire, Rosen, & Botvinick, 2010), observers may rely on a single attentional control process instead of maintaining two simultaneous processes, as suggested by Chang and Egeth (2019), in the performance of visual search. Thus, it would be valuable to gain further insights into the mechanisms of attentional enhancement and distractor inhibition.

In the present study, we first repeated the work of Chang and Egeth (2019) with the intention of replicating their findings. We then modified their paradigm to re-investigate whether target enhancement and distractor suppression can guide attention independently. Experiment 1 was drawn from Chang and Egeth (2019) and was performed as an online experiment. In addition to group-level analyses, as performed in their work, we explored differences in behavior among individuals. Specifically, we investigated a correlation between target enhancement and the effects of distractor suppression. We obtained the raw data of Chang and Egeth (2019) and applied the same analysis, hypothesizing that if target enhancement and distractor suppression could guide attention independently, as Chang and Egeth (2019) argued, no negative correlation would be observed. Furthermore, Experiments 2 and 3 were intended to quantify the effects of target enhancement and distractor suppression separately by manipulating color combinations of the target and the distractor. In particular, unlike the use of fixed colors for the search target and distractor in Experiment 1, we altered the search target and distractor colors on a trial-by-trial basis in Experiments 2 and 3, respectively. Thus, the participants could expect only a target or a distractor color. Accordingly, we calculated the magnitude of the enhancement and suppression for each participant and compared the effect across experiments. We hypothesized that if two attentional-guidance elements, enhancement and suppression, competed for common processing resources, the magnitude of the effect in Experiment 1 would be smaller than those in Experiments 2 and 3 because Experiment 1 required both of these attentional controls.

## Methods

### Study design

**Figure 1.**
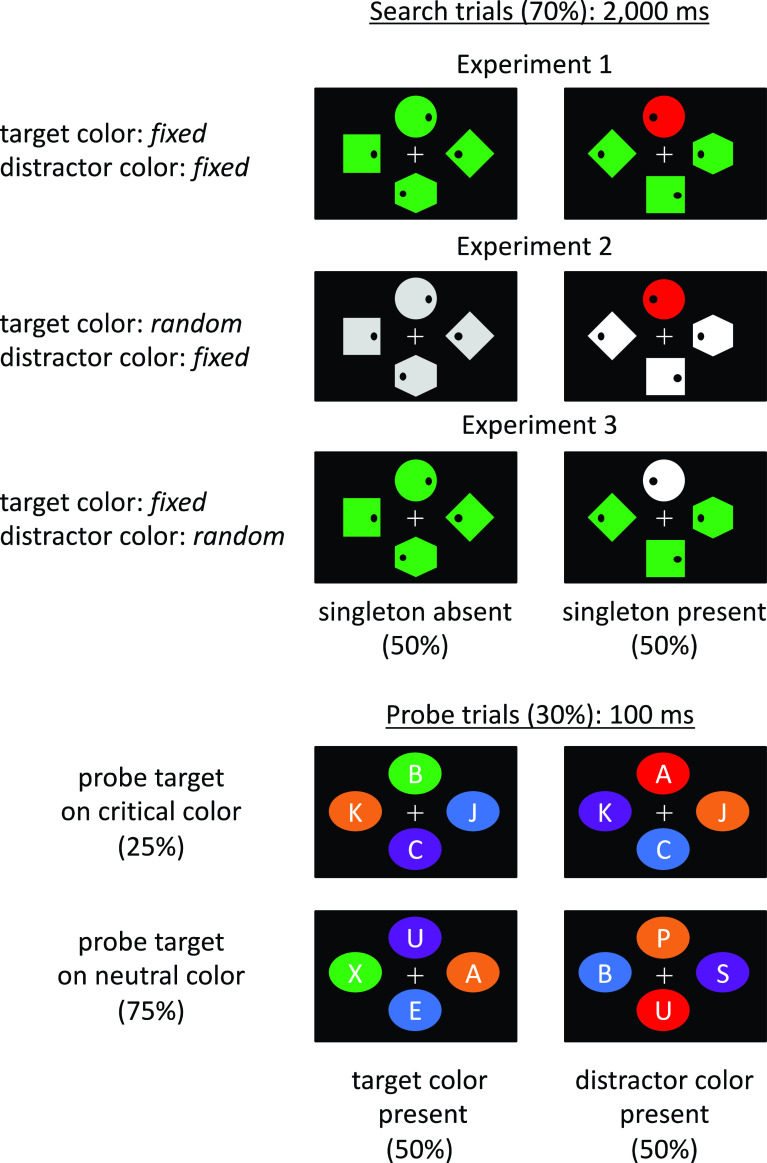
Schematic illustration of experimental trials. In the search trials (top panel), participants were asked to report whether a dot inside the search target (diamond) was presented on the left or right. In half of the trials, a different color (singleton) was presented. The target color was fixed (Experiments 1 and 3) or random (Experiment 2); the distractor color was also fixed (Experiments 1 and 2) or random (Experiment 3). In the probe trials (bottom panel), the task was to detect a probe target (A or B). In Experiment 1, in half of the trials, the target color (e.g., green) was presented, while in the other half, the distractor color (e.g., red) was presented. The probe target appeared in a critical color (either of the target or distractor color in search trials) or in a neutral color (a color that had not been presented in search trials). In Experiments 2 and 3, either the distractor or target color appeared in half of the probe trials, respectively, while all items were presented in neutral colors in the other half of the trials.

Schematic illustrations of experimental trials are shown in Figure 1. Search trials (70% of all trials) and probe trials (30%) were randomly presented to participants. In the search trials, participants were asked to report whether a dot inside the search target (diamond) was presented on the left or right. In the probe trials, participants were required to detect a probe target (A or B). The probe target appeared in a critical color (either of the target or distractor color in search trials) or in a neutral color (a color that had not been presented in search trials). Experimental codes can be found at https://doi.org/10.5281/zenodo.5944534 ( Kawashima & Amano, 2022a).

### Participants

The research subjects were healthy adults between the ages of 20 and 35 living in Japan from the Center for Information and Neural Networks (CiNet)’s research participation pool. In total, 150 participants were enrolled in this study via the web-based SONA recruitment system ( www.sona-systems.com) and were promised monetary compensation (1,000 Japanese Yen or approximately 10 US dollars). Three experiments were conducted, each with 50 different participants. The color combinations for the target and distractor differed across the three experiments. A minimum sample size of 25 was estimated via a priori power analysis using G*Power ( Faul, Erdfelder, Buchner, & Lang, 2009: with settings of power = 0.80, alpha = 0.05, and $\eta_{p}^{2}$ = .374) based on a previous report ( Chang & Egeth, 2019). We doubled this number because of the possible large variability inherent in data collected through online experimentation.

In Experiment 1, four participants were removed owing to their probe task performance, which was below the level of chance. Ultimately, 46 participants, who were aged between 20 and 30 years ( *M* = 22.3, *SD* = 2.04, 17 female participants), were included in the analyses. For the same reason, two and three participants were removed from Experiments 2 and 3, thereby resulting in 48 ( *M* = 21.9, *SD* = 1.36, 26 female participants) and 47 ( *M* = 22.8, *SD* = 2.63, 22 female participants) total participants, respectively.

### Ethics and consent

The study was approved by the ethics and safety committee of the National Institute of Information and Communications Technology in Osaka, Japan (approval number: 20191031). Informed consent was obtained from all participants. The informed consent statement was displayed on the monitor at the beginning of the online experiment, with a full description of the study purpose, authors identification, and that data would be stored privately with authors. Participants expressed their willingness to participate in the experiment by pressing a predetermined key. All methods were performed in accordance with the relevant guidelines and regulations.

### Data collection

All experiments were performed online. Participants obtained the link to the online experiment through the SONA Systems recruitment website and, after clicking on the link, the experiment started. Participants were asked to participate in the experiment in a quiet room and not to use cell phones or listen to music during the experiment.

**Stimuli and experiment design**

Stimuli were generated and presented via Pavlovia.org based on PsychoPy (v2020.1: Peirce *et al.*, 2019). At the beginning of the experiment, we calculated the pixel density (pixel/mm) of the participants’ monitors using a card task and then calculated their viewing distance with a blind spot task ( Li, Joo, Yeatman, & Reinecke, 2020). The blind spot task featured three practice trials (repeated if needed), followed by five experimental trials. The same five trials of the blind spot task were presented in each half of the main task to obtain a reliable viewing distance throughout the experimental session. These parameters were used to determine the visual angle of the stimuli.

We created search and probe displays similar to those of Chang and Egeth (2019), as shown in Figure 1. The search (70% of trials) and probe trials (30% of trials) were presented to the participants randomly. The search and probe displays contained four geometric shapes presented at each corner of an imaginary diamond with a diagonal of 10.34°. The search displays contained one diamond, one circle, one square, and one hexagon (1.7° × 1.7°). The search target was the diamond. Each shape contained a black dot (0.15°) located 0.3° to the right or left side of the shape. The task was to report the dot location of the target shape by pressing the “F” or “J” keys for the left or right side, respectively, as quickly and accurately as possible. For 50% of the search trials, one randomly chosen distractor item was presented as a singleton (i.e., one shape was drawn in a different color). This point was explicitly mentioned in the instruction. In the remaining trials, no singleton was presented (i.e., all the shapes were drawn in the same color). The location of the target dot, target shape, and singleton varied randomly. The search trials began with a black screen (500 ms) followed by a fixation cross (800 ms) and then a search display (2,000 ms). A feedback display was subsequently presented for 500 ms with the word “correct!” or “error” based on the participants’ responses.

The target and distractor colors were fixed in the task in Experiment 1. These colors were assigned to the participants randomly. In Experiment 2, only the distractor color was fixed throughout the trials; the target color was varied randomly on a trial-by-trial basis. In Experiment 3, only the target color was fixed; the distractor color was changed for each trial.

The probe displays contained four different colored ovals (1.5° × 1.2°), inside which a letter (0.75° in height) was presented. The task was to detect the letter A or B in the display and to press F or J keys in response, respectively. The other three letters were randomly selected from other alphabets, with the exception of I and O. The probe trials began with a black screen (500 ms) and the subsequent fixation cross (800 ms) followed by a probe display for 100 ms. The observers had to press a key within 3,000 ms after the presentation of the probe. A feedback display was then presented for 500 ms with the word “correct!” or “error” based on the participant’s key press.

The target–distractor color combinations were manipulated in the experiments. For Experiment 1, 50% of the probe trials contained the target color in the search trials, where the target color that appeared in the search trials also appeared in the probe trials (i.e., the target *-*color *-*present trials). The probe target was presented on the target-colored items in 25% of this group of trials or on neutral-colored items in 75% of this group. The remaining 50% of probe trials included a distractor color in the search trials (i.e., the distractor-color-present trials) where the probe target appeared on a distractor-colored item in 25% of trials or on a neutral-colored item in 75% of trials. The location of the probe target was varied randomly. For Experiment 2, all the target-color-present trials in Experiment 1 were replaced with neutral trials where all probes were colored in neutral colors. Thus, the rate of distractor-color-present trials was maintained at the same level as that of Experiment 1. Namely, 50% of probe trials included a distractor color (25% featured a probe on a distractor-colored item, and 75% featured a probe on neutral-colored items). Conversely, the remaining 50% of probe trials included neutral-colored items. Similarly, for Experiment 3, 50% of the probe trials included the target color (25% had a probe on the target-colored item, and 75% had a probe on the neutral-colored items), and the remainder included neutral-colored items.

Five distinctive colors (red, medium blue, forest green, dark orange, and magenta) were used as the target, distractor, and neutral colors. Their roles were randomly assigned across participants. Four other unique colors (white, light gray, dark gray, and dim gray) were used as the target color (Experiment 2) and distractor color (Experiment 3), respectively. We used grayscale as random colors to avoid potential confounds in the color space associated with fixed colors; target-to-target and distractor-to-distractor color similarity could affect the formation of the search template ( Geng & Witkowski, 2019). The experiment was preceded by 10 practice trials for the search task and 10 practice trials for the probe task. The main experiment comprised 448 search trials and 192 probe trials (640 trials in total) with an opportunity given for a brief rest every 40 trials.

### Analysis

All data analysis was carried out using R (version 4.0.3). In the search task, a paired *t*-test was used to compare the reaction times (RTs) between singleton-present and singleton-absent trials. In the probe task, we conducted repeated-measures analysis of variance (ANOVA) with within-subject factor critical color and within-subject factor probe-target location on RTs and accuracy (Experiment 1). For Experiment 2 and 3, a paired *t*-test was used to compare the RTs and accuracy between probe conditions. A Pearson correlation analysis was conducted for evaluating the relationship between target enhancement and distractor suppression. Target enhancement effect was calculated by subtracting the RTs for critical-colored condition (probe on a target color) from those for neutral-colored condition, while distractor suppression effect was calculated by subtracting the RTs for critical-colored condition (probe on a distractor color) from those for neutral-colored condition.

**Re-analysis of Chang and Egeth (2019)**

We obtained the raw data from Chang and Egeth (2019) and applied the same correlation analyses as those used in Experiment 1. Specifically, to assess the relationship between target enhancement and distractor suppression, we calculated the magnitude of enhancement by subtracting the RTs on target-color trials from those on neutral-color trials and the magnitude of suppression by subtracting the RTs in neutral-color trials from those in distractor-color trials. Furthermore, we combined correlation coefficients of those in Experiment 1 and in Chang and Egeth (2019) to better ascertain whether enhancement and suppression could guide attention independently.

## Results

### Search task

Trials with RTs faster than 100 ms or slower than 5,000 ms were excluded from the analysis. Further, we removed trials with RTs 3.5 standard deviations above or below the mean for each participant, resulting in the elimination of 0.4%, 0.6%, and 0.5% of all the search trials in Experiments 1, 2, and 3, respectively. Full raw data for all the experiments can be found under *Underlying data* ( Kawashima & Amano, 2022b).

**Figure 2.**
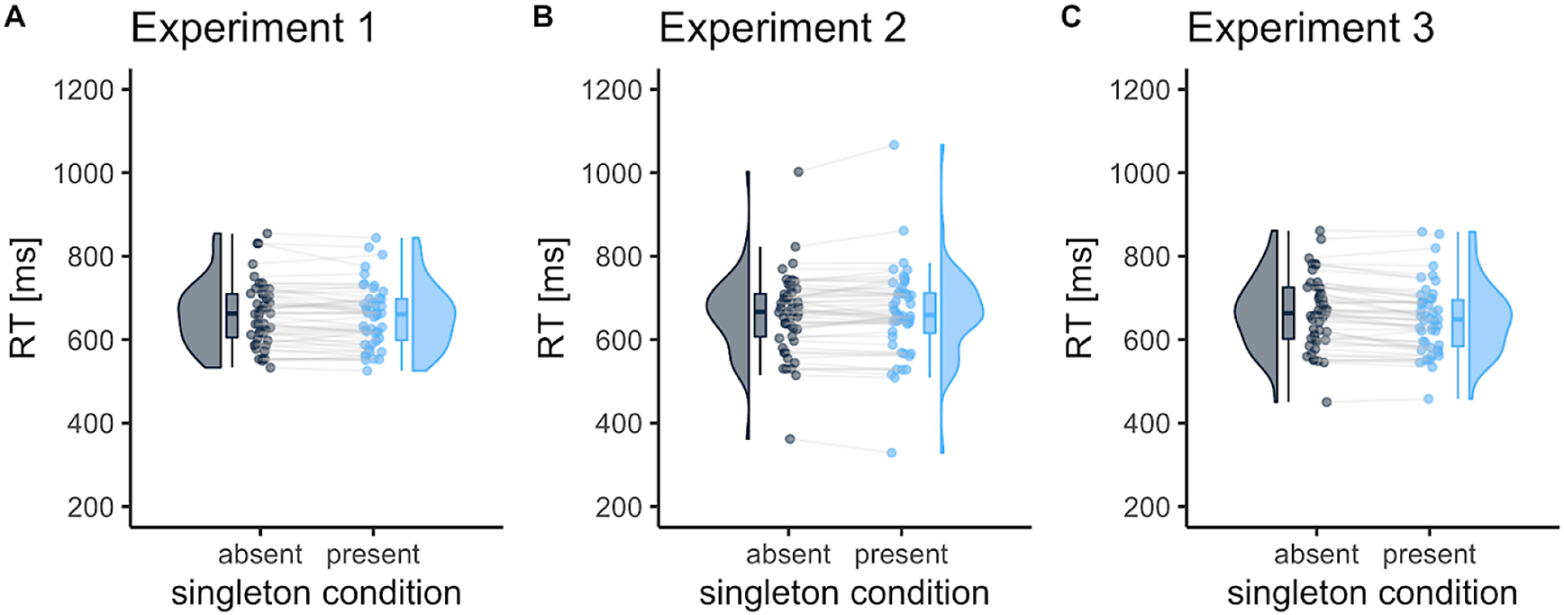
The mean reaction times (RTs) in the visual search task as a function of the singleton condition. Each dot represents the mean RT per participant. Error bars indicate standard errors of the mean. The RT was shorter in singleton-present trials than in singleton-absent trials for Experiments 1 and 3.

All participants performed well on the search task ( *n* = 46, 48, and 47 for Experiment 1, 2, and 3, respectively). The mean accuracy was 96.5% (95.5%, 96.2%) and 96.9% (95.3%, 96.5%) for singleton-present and singleton-absent trials in Experiments 1, 2, and 3, respectively. Therefore, we did not examine accuracy owing to possible ceiling effects. A pairwise *t*-test was used to compare the RTs between the trials. Figure 2 shows the mean RTs in the visual search task. In Experiment 1, where target and distractor colors were fixed during the trials, the RTs were faster for singleton-present trials than in singleton-absent trials (−5.8 ms [95% CI, −0.23 to −11.3]; *t* (45) = 2.10, *p* = .041, *d* = 0.31). This singleton-presence benefit fits with earlier findings [8,10], allowing us to test target enhancement and distractor suppression for probe trials. Unexpectedly, however, in Experiment 2, where the target colors were randomized and the distractor color was fixed, no RT differences were observed between singleton-present and singleton-absent trials (2.8 ms [95%CI, −3.2 to 8.8]; *t* (47) = 0.94, *p* = .354, *d* = 0.14). However, in Experiment 3, where the target color was fixed and the distractor colors were random, singleton-present benefits were observed in the RTs (−14.0 ms [95% CI, −8.6 to −19.4]; *t* (46) = 5.20, *p* < .001, *d* = 0.76).

### Probe task

The same criteria were used to exclude trials in search tasks. We removed trials if two or more consecutive preceding trials were probe trials, because successive probe tasks could transiently disrupt the attentional set for target or distractor and thus distort probe responses ( Chang & Egeth, 2019; Gaspelin & Luck, 2018a), which resulted in the elimination of 9.8%, 9.7%, and 10.0% of all probe trials in Experiments 1, 2, and 3, respectively. We performed a 2 (critical color: target color or distractor color presented on a probe trial) × 2 (probe-target location: neutral-colored or critical-colored item on which the target was present) repeated-measures analysis of variance for both RT and accuracy. Please note that target enhancement effect is calculated by subtracting the RTs for critical-colored condition (probe on a target color) from those for neutral-colored condition, while distractor suppression effect is calculated by subtracting the RTs for critical-colored condition (probe on a distractor color) from those for neutral-colored condition.

**Figure 3.**
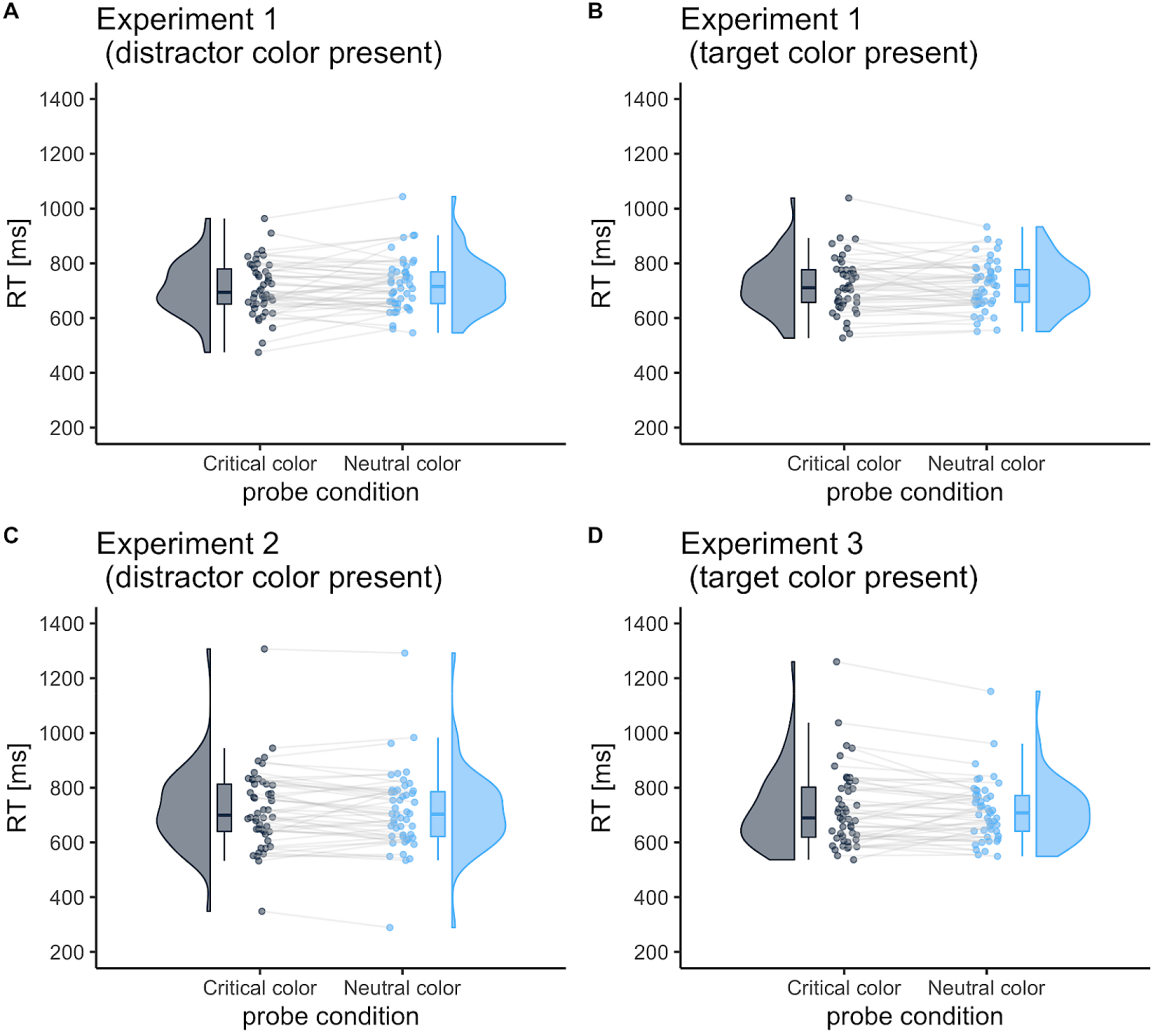
Mean reaction time (RT) in the probe task as a function of probe-target color. The probe-target color was either the critical color or neutral color. The critical color was either the distractor or target color in Experiment 1; it was always the distractor color in Experiment 2 or the target color in Experiment 3. The neutral color was one that had not been presented in search trials. Each dot represents the mean RT per participant.

In Experiment 1 ( Figure 3A and B), no significant main effect of critical color and probe-target location on RT was found ( *F* (1, 45) = 1.00, *p* = .321, $\eta_{p}^{2}$ = .02; *F* (1, 45) = 0.53, *p* = .471, $\eta_{p}^{2}$ = .01), nor was there any significant interaction ( *F* (1, 45) = 2.37, *p* = .130, $\eta_{p}^{2}$ = .05). These results indicate no enhancement or suppression effects in the probe task (−13.4 ms [95% CI, −6.9 to −33.9]; −1.7 ms [95%CI, −19.4 to 16.0]: the scatter plot in Figure 5B). This remained true even when the target or distractor colors were presented randomly in the visual search task (Experiments 2 and 3): no difference was observed in the probe location for the suppression effect ( Figure 3C: 8.5 ms [95%CI, −8.4 to 25.3]; *t* (47) = 1.01, *p* = .318, *d* = 0.15: the scatter plot in Figure 5A) and for the enhancement effect ( Figure 3D: −8.1 ms [95%CI, −27.4 to 11.1]; *t* (46) = 0.85, *p* = .400, *d* = 0.12: the scatter plot in Figure 5C).

**Figure 4.**
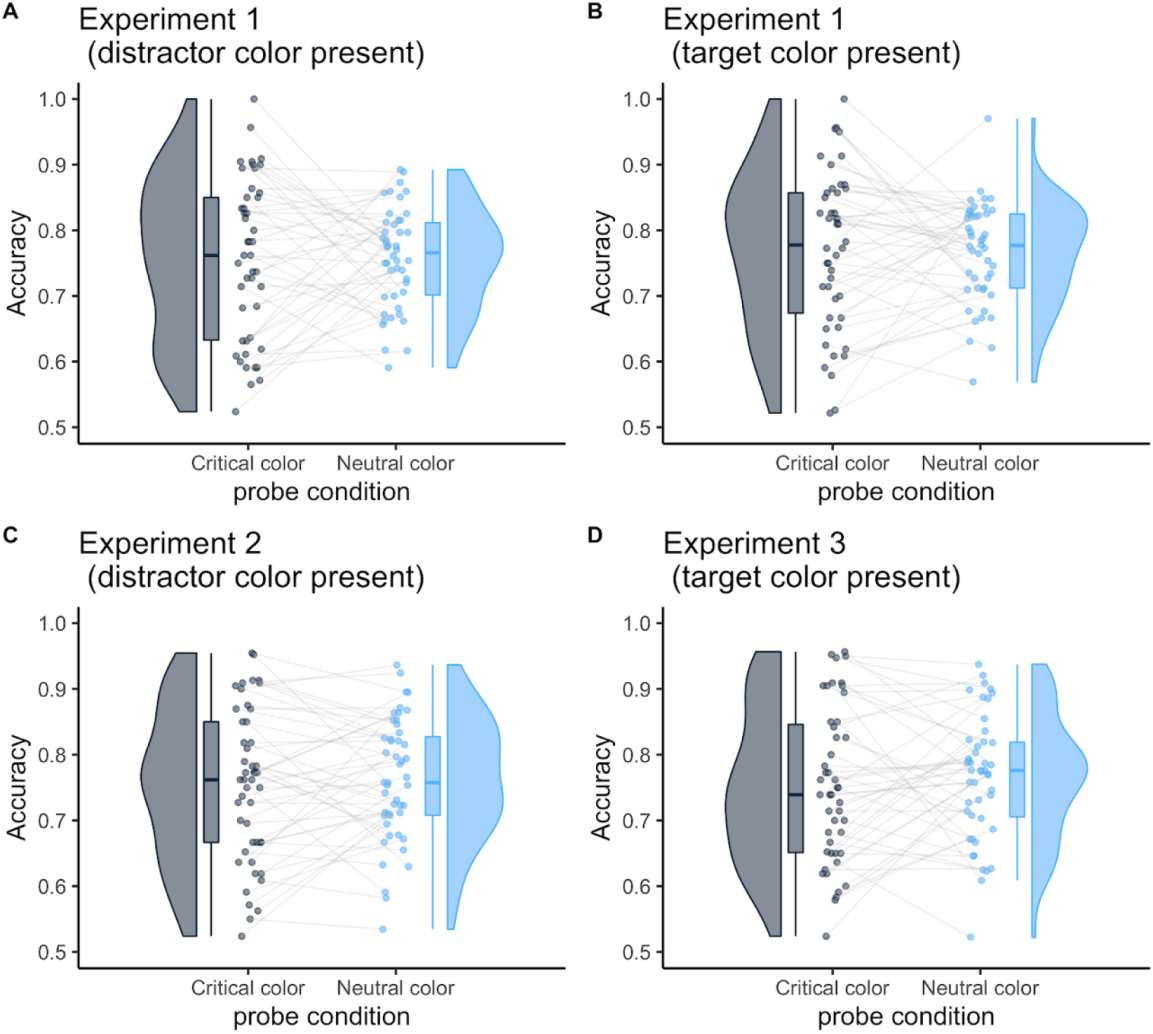
Mean accuracy in the probe task as a function of probe-target color. The probe-target color was either the critical color or neutral color. The critical color was either the distractor or the target color in Experiment 1; it was always the distractor color in Experiment 2 or the target color in Experiment 3. The neutral color was one that had not been presented in search trials. Each dot represents the mean accuracy per participant.

For accuracy, in Experiment 1 ( Figure 4A and B), there was neither a significant main effect of critical color and probe-target location ( *F* (1, 45) = 1.00, *p* = .323, $\eta_{p}^{2}$ = .02; *F* (1, 45) = 0.0002, *p* = .989, $\eta_{p}^{2}$ = .00) nor a significant interaction ( *F* (1, 45) = 0.06, *p* = .810, $\eta_{p}^{2}$ = .00). These results indicate no enhancement or suppression effects in the probe task (−1.7 % [95% CI, −19.4 to 16.0]; −13.5 % [95%CI, −33.9 to 6.9]). In addition, we found no difference in the probe location in Experiments 2 and 3 (the suppression effect: Figure 4C, −0.008 % [95%CI, −0.04 to 0.02]; *t* (47) = 0.60, *p* = .551, *d* = 0.09; the enhancement effect: Figure 4D, 0.01% [95%CI, −0.02 to 0.05]; *t* (46) = 0.75, *p* = .455, *d* = 0.11).

**Figure 5.**
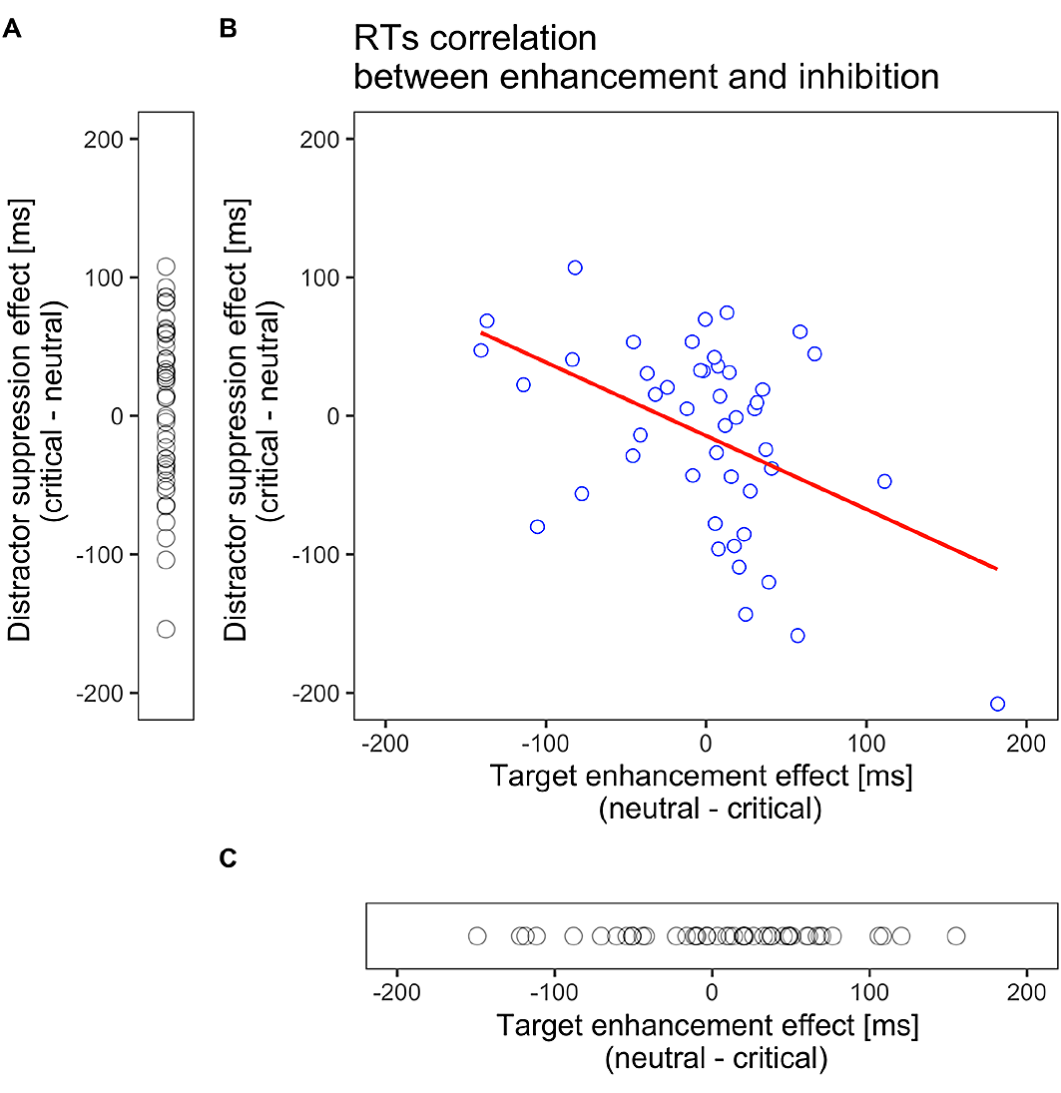
Scatter plots for target enhancement or distractor suppression. (A). Probe effect of all subjects in Experiment 2. Distractor suppression effect varied across subjects, and no group-level effect was observed. (B). Correlations between target enhancement and distractor suppression effects in Experiment 1 for reaction times (RTs). Target enhancement (distractor suppression) indicates a faster RT for the target-colored probe than the neutral probe, while distractor suppression indicates a faster RT for the distractor-colored probe than the neutral probe. The red line indicates the best fit line to the data. The target enhancement and distractor suppression were negatively correlated (Pearson’s *r* = −.46, *p* = .001), suggesting that they are not mutually independent. (C) Probe effect of all subjects in Experiment 3. Target enhancement effect varied across subjects, and no group-level effect was observed.

Our results in Experiment 1 showed no enhancement or suppression effects in either RT or accuracy in the probe task, which does not support the findings of Chang and Egeth (2019). This may be due to the larger variability across participants in search performances. Therefore, we assessed the relationship between target enhancement and distractor suppression effects in Experiment 1. The magnitude of enhancement was calculated by subtracting the RTs on target-color trials from those on neutral-color trials. The magnitude of suppression was obtained by subtracting RTs in neutral-color trials from those in distractor-color trials. As shown in Figure 5B, we found a significant negative correlation between them ( *r* = −.46, 95% CI [–.66, –.20], *p* = .001, *t* (44) = 3.44). This negative correlation remained significant after Spearman’s rank-order correlations, which are less sensitive to outliers than Pearson’s product-moment correlation ( *r*s = −.40, *p* = .006). This correlation indicates that those with larger RT benefits by target enhancement had less inhibition to the distractor color and vice versa.

**Figure 6.**
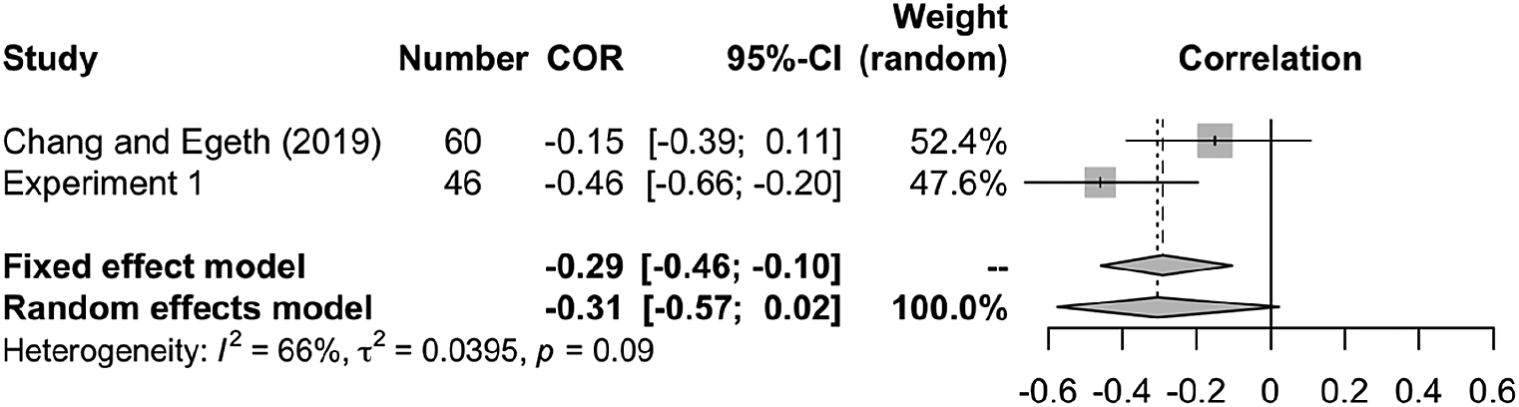
Meta-analysis of studies on the correlation between target enhancement and distractor suppression. Forest plot indicates pooled individual-study Pearson’s correlation coefficients with corresponding 95% CIs. Random effect model showed a marginal negative correlation between target enhancement and distractor suppression ( *r* = −.31, 95% CI [−.57, .02], *p* = .068), suggesting that they are not mutually independent.

To improve the comparison of our findings to those of Chang and Egeth (2019), we obtained their raw data and applied the same correlation analyses. We found a numerically negative but nonsignificant correlation between target enhancement and distractor suppression ( *r* = −.15, 95% CI [−.39, .11], *p* = .257, *t* (58) = 1.15). Further, we applied a meta-analysis of correlations between Chang and Egeth (2019) and Experiment 1 of the present study using the “meta” package in R ( Schwarzer, 2007). As Figure 6 shows, the pooled correlation in this dataset is *r* = −.31 (95% CI [−.57, .02], for the random-effects model), which is marginally significant ( *z* = −1.83, *p* = .068).

Although a negative correlation between the magnitude of distractor suppression and attentional enhancement was found for RT, no correlation was found for accuracy in Experiment 1 ( *r* = −.13, 95% CI [−.40, .16], *p* = .378, *t* (44) = 0.89) and in Chang and Egeth’s (2019) data ( *r* = .16, 95% CI [−.09, .40], *p* = .211, *t* (58) = 1.27). We checked for a possible speed-accuracy trade-off in Experiment 1, analyzing RT and accuracy together by computing inverse efficiency scores (RT/proportion correct: Bruyer & Brysbaert, 2011) for each participant and condition in Experiments 1, 2, and 3. The inverse efficiency score combines both RT and accuracy into a single measure. The ANOVA of the inverse efficiency index in Experiment 1 also revealed a non-significant probe effect reflected in no interaction between critical color and probe-target location ( *F* (1, 45) = 0.12, *p* = .733, $\eta_{p}^{2}$ = .003). Neither was any probe effect observed in Experiments 2 and 3 ( *t* (47) = 1.32, *p* = .194, *d* = 0.19; *t* (46) = 1.29, *p* = .204, *d* = 0.19). This lack of probe effect in inverse efficiency scores indicates that faster RTs were not accompanied by a sharp decrease in accuracy in our task.

## Discussion

The present study tested whether target enhancement and distractor suppression can work independently. We observed the singleton-present benefit of RTs in the visual search task, replicating previous findings ( Chang & Egeth, 2019, 2021; Gaspelin & Luck, 2018a). This suggests that a salient distractor in a visual display can be excluded from selection, supporting the idea of a signal-suppression hypothesis ( Sawaki & Luck, 2010, 2013). However, the RT benefits in visual search unexpectedly disappeared when the target color was varied from trial to trial (Experiment 2; Figure 2B). One possible reason for the lack of RT benefits is that, to some extent, the participants relied on attentional enhancement to target items rather than direct suppression to exclude the salient item. In Experiment 2, the participants could not expect target features due to its randomness, so the contribution of attentional enhancement in the visual display was lower than that in Experiment 1 and 3. This makes it plausible that target enhancement guides visual attention more effectively than distractor suppression.

We found a singleton-presence benefit in Experiment 3, where the distractor color varied on a trial-by-trial basis. However, this observation seems to be inconsistent with previous results suggesting that singletons are suppressed based on their color dimension (first-order suppression: Gaspelin & Luck, 2018a). Vatterott and Vecera (2012) asked participants to perform a visual search task where singleton color was constant for block 1 (48 trials) before changing to a different color in block 2. These authors found that the singleton captured the participants’ attention (singleton-presence cost) in the first half of block 1, while this cost was eliminated (singleton-presence benefit) in the second half of block 1, indicating that the participants learned to suppress the salient item (see also Vatterott, Mozer, & Vecera, 2018). Notably, the singleton-presence cost was observed again in the first half of block 2, where the singleton color was changed. Similarly, Gaspelin and Luck (2018a) demonstrated that oculomotor suppression effects ( Gaspelin, Leonard, & Luck, 2017) were reduced when the singleton color was changed, suggesting that first-order feature suppression plays a crucial role. However, our results show that even when the singleton color was changed from trial to trial, a singleton-presence benefit was observed for the RTs ( Figure 2C). One difference between the previous studies and ours is the frequency of changes in the singleton colors on a blocked or trial-by-trial basis. Following the observation that attentional capture by distractors can be suppressed by increasing repetitions (repetition suppression: Bonetti & Turatto, 2019; Thompson, 2009), our frequent changes in singleton color may have prevented this habituation-based inhibition, instead promoting a conceptual suppression of the singleton. Thus, our finding of a changing-color singleton-presence benefit suggests that singletons can be suppressed based not only on color information but also on a higher conceptual level of information, such as the semantic levels of the description of saliency (second-order suppression: Gaspelin & Luck, 2018a). This second-order suppression has also been reported in the domain of spatial attention ( Won, Kosoyan, & Geng, 2019; Won, Forloines, Zhou, & Geng, 2020).

The participant-level analysis presented in Figure 5B shows a negative correlation between the RT effects of target enhancement and distractor suppression. This result does not support the idea of concurrent attentional guidance through enhancement and suppression proposed by Chang and Egeth (2019). Rather, this negative correlation indicates that the two are not independent. Combining the observations that the distributions of target enhancement and distractor suppression effects in Experiment 1 ( Figure 5B) resembled those in Experiments 2 ( Figure 5A) and 3 ( Figure 5C), the results indicate that participants encountered difficulties in using the two representations for enhancement and suppression—that is, whether attentional enhancement or distractor suppression works depends on the observer’s choice of search strategy. Some participants would cease suppressing irrelevant distractors because of the effort required: an empirical study showed that participants selectively encode positive information for visual search even when both positive and negative cues are provided ( Rajsic *et al.*, 2020). Thus, those who attempt to focus more on the target dimension would be more drawn to the distractor and vice versa.

This difference in search strategy across individuals may be one reason why no attentional enhancement or distractor suppression was observed in group-level analysis. Although a singleton-presence benefit of RTs was observed in visual search, we found no target enhancement or distractor suppression in the probe task in Experiment 1 ( Figures 3A and 4A). Thus, we failed to replicate the previous findings of Chang and Egeth (2019), thereby casting doubt on their claim that attention can be guided concurrently by enhancement and suppression. Here, we propose instead that differences in search strategy among the participants might have made group-level effects invisible.

Chang and Egeth’s (2019) data showed a numerically negative but nonsignificant correlation between target enhancement and distractor suppression. This was in contrast to our argument that participants select and rely on a single search strategy to perform the search task. Why were we unable to find group-level effects for enhancement and suppression, unlike Chang and Egeth (2019)? It is possible that our failure of replication was due to our less controlled setting—a result of the online constraints of our experiments. However, we believe that this account is incomplete because we replicated the singleton-presence benefit in visual search in experimental online settings, showing the precise measurements for RT (e.g., 5.7 ms benefit in Experiment 1). In addition, we found no probe effect in inverse efficiency scores, indicating that enhancement and suppression effects were still not observed even when a larger variability in accuracy was incorporated. Thus, we believe that a potential lack of complete engagement by the participants in the task because of the online setting cannot fully explain our results. Another possible reason is differences in instruction. Previous studies have shown that an awareness of distractors modulates the interference of the distractor ( Chisholm & Kingstone, 2014: Huffman, Rajsic, & Pratt, 2019). Chisholm and Kingstone (2014) assessed the influence of awareness on attentional capture by informing some participants of the presence of the distractor (aware condition) and asking others to avoid attending to the distractor (avoid condition). Their results showed that the oculomotor capture of the distractor in the avoid condition was larger than that in the aware condition, suggesting that too much of an emphasis on distractor suppression could lead to a larger interference. Based on these findings, the slight differences in instructions between Chang and Egeth (2019) and our study might have resulted in the observed inconsistency in the results. For our online experiment, the instruction was presented as screen text, and understanding it was entirely dependent on the participants. For onsite experiments, as in Chang and Egeth (2019), generally, the instructions can be repeated several times, and their emphasis is dependent on the experimenter. Although this attribution is speculative, such a difference in instruction could yield a different attentional set to the task, which may be a reason for the failure of replication. Future research is required to control the attentional set through the instructions for exploring the mechanism of distractor suppression, particularly regarding the comparison of online and offline results. However, another possibility concerns methodological differences. Chang and Egeth (2019) gave 32 probe and 48 search practice trials; to save time, we gave 10 probe and search practice trials. These differences in the exposure to target and distractor prior to the task might have led to the weak enhancement and suppression in the current study. Future work should consider the role of practice in the mechanism of attentional guidance. Finally, the limitation of this study is that the sample size was rather small for observing the correlation between target enhancement and distractor suppression. Although the combined correlation coefficients of those in Experiment 1 and in Chang and Egeth (2019) showed a negative trend ( Figure 6), future studies with a larger sample size and controlled settings might confirm our findings.

Our current results constitute a contribution to the understanding of how target enhancement and distractor suppression are coordinated to allocate visual attention. Some have suggested that the two operate concurrently to guide visual attention ( Andersen & Müller, 2010; Navalpakkam & Itti, 2007), leading to the idea of an enhancement-and-suppression model ( Chang & Egeth, 2019). Our results, however, show that, instead of attentional guidance occurring independently through enhancement and suppression, it depends on the participants’ search strategy: some use target enhancement; others use distractor suppression for the guidance of visual attention. These findings suggest that maintaining these two control systems simultaneously would be cognitively demanding. Future research should examine the contributions of explicit learning (e.g., strategy choice) and implicit learning (e.g., excessive training) in suppressing distractors ( Luck, Gaspelin, Folk, Remington, & Theeuwes, 2021).

## Data availability

### Underlying data

OSF: Can enhancement and suppression concurrently guide attention? An assessment at the individual level. https://osf.io/bpz3k ( Kawashima & Amano, 2022b)

This project contains the following underlying data:

- raw_data.xlsx (Raw data from search trials and probe trials)

### Extended data

This project contains the following extended data:

- Experiment files (JavaScript code for each Experiment 1, 2, and 3)

Data are available under the terms of the Creative Commons Attribution 4.0 International license (CC-BY 4.0).

## Software availability

Source code available from:

Experiment 1 ( https://gitlab.pavlovia.org/Kawashima/exp1_kawashima_amano)

Experiment 2 ( https://gitlab.pavlovia.org/Kawashima/exp2_kawashima_amano)

Experiment 3 ( https://gitlab.pavlovia.org/Kawashima/exp3_kawashima_amano)

Archived source code at time of publication: https://doi.org/10.5281/zenodo.5944534 ( Kawashima & Amano, 2022a)

**License:** GNU General Public License, version 3

## Author contributions

T.K. performed experiments and analyzed data; and K.A. and T.K. designed experiments, defined and validated data analysis methods, and wrote the paper.
