# Supplementary material for: Can enhancement and suppression concurrently guide attention? An assessment at the individual level
Source: F1000Res. 2022 Sep 26;11:232. Originally published 2022 Feb 24. [Version 2] doi: 10.12688/f1000research.77430.2 (PMC9237560; doi:10.12688/f1000research.77430.2)
Supplement: Peer review history

# Peer review: Reviewer response for version 2

**DOI:** 10.5256/f1000research.138730.r151578

**Published:** 2022-10-19

**Authors:** Ru-Yuan Zhang

**Affiliations:** 1 Institute of Psychology and Behavioral Science, Shanghai Jiao Tong University, Shanghai, China

I think the current version is substantially improved. I am OK with the publication of this paper.

Is the work clearly and accurately presented and does it cite the current literature?

Yes

If applicable, is the statistical analysis and its interpretation appropriate?

Partly

Are all the source data underlying the results available to ensure full reproducibility?

Yes

Is the study design appropriate and is the work technically sound?

Partly

Are the conclusions drawn adequately supported by the results?

Yes

Are sufficient details of methods and analysis provided to allow replication by others?

Yes

Reviewer Expertise:

Visual perception, cognitive neuroscience

I confirm that I have read this submission and believe that I have an appropriate level of expertise to confirm that it is of an acceptable scientific standard.

# Peer review: Reviewer response for version 1

**DOI:** 10.5256/f1000research.81418.r140483

**Published:** 2022-06-30

**Authors:** Ru-Yuan Zhang

**Affiliations:** 1 Institute of Psychology and Behavioral Science, Shanghai Jiao Tong University, Shanghai, China

**Summary:**

 This study focuses on the relative contributions of target enhancement and distractor suppression in visual search. In particular, the authors designed three experiments. In Experiment 1, the authors attempted to replicate the singleton-presence benefits as reported in Chang & Egeth, 2019 and found negative results. In Experiments 2 and 3, the randomness of target colors and distractor colors in the search task was systematically manipulated. However, these manipulations seem to exert no effects on reaction time and accuracy. Most importantly, the authors independently calculated the target enhancement effect and the distractor suppression effect (i.e., RT differences as compared to the neural conditions) and discovered a negative correlation between the two. This novel negative correlation suggests target enhancement and distractor suppression may act as the two sides of the same coin—the shared cognitive mechanisms.

 In general, I like the overall experimental design and the arguments here, albeit the majority of the results being negative. I especially appreciate the authors’ efforts to replicate a published study and extend it. We should encourage reporting negative and replication results. I have several comments as below that may help improve the manuscript.

**Major points:**

1. The experimental design is OK, but the descriptions of the experimental details are hard to follow. Fig.1 is particularly confusing, and I have to read line-by-line in the methods part in a combination of Fig. 1 to comprehend what exactly the authors meant. I suggest substantially improving Fig.1 and the figure caption underneath. 
2. Reaction time is usually highly skewed distributed. It seems to me that the authors performed stats on the raw RT values. I suggest trying to perform stats on log-transformed RT. That should better fit the intentions of t-tests and ANOVAs.
3. It remains unclear to me the rationales behind Experiments 2 and 3. The authors mentioned that “We hypothesized that if two attentional- guidance elements, enhancement and suppression, competed for common processing resources, the magnitude of the effect in Experiment 1 would be smaller than those in Experiments 2 and 3 because Experiment 1 required both of these attentional controls.” This argument is unclear to me. If either the target or distractor color is randomly selected, the advantage of color expectation should be diminished and that would lead to a smaller enhancement or suppression effect. Can the authors elaborate on the rationales and the predictions?

**Minor points:**

1. “This singleton-presence benefit fits with earlier findings [8,10], allowing us to test target enhancement and distractor suppression for probe trials. ” Here the numbered-style references should be replaced by inline-style references.
2. The figure captions should be improved. For example, in Fig. 2, does the black horizontal line in the box plot represent the mean or median of the group? What are the definitions of the upper and lower edges of the boxes and of the range of the whiskers? 

Is the work clearly and accurately presented and does it cite the current literature?

Yes

If applicable, is the statistical analysis and its interpretation appropriate?

Partly

Are all the source data underlying the results available to ensure full reproducibility?

Yes

Is the study design appropriate and is the work technically sound?

Partly

Are the conclusions drawn adequately supported by the results?

Yes

Are sufficient details of methods and analysis provided to allow replication by others?

Yes

Reviewer Expertise:

Visual perception, cognitive neuroscience

I confirm that I have read this submission and believe that I have an appropriate level of expertise to confirm that it is of an acceptable scientific standard, however I have significant reservations, as outlined above.

# Response

**Published:** 2022-09-16

**Authors:** Tomoya Kawashima

- The experimental design is OK, but the descriptions of the experimental details are hard to follow. Fig.1 is particularly confusing, and I have to read line-by-line in the methods part in a combination of Fig. 1 to comprehend what exactly the authors meant. I suggest substantially improving Fig.1 and the figure caption underneath.

Thank you for the suggestion. We amended Figure 1 and added a few statements in the caption to further illustrate the experimental design.

**Figure 1. Schematic illustration of experimental trials. **The search (70% of trials) and probe trials (30% of trials) were presented to the participants randomly. In the search trials (top panel), participants were asked to report whether a dot inside the search target (diamond) was presented on the left or right. The four stimuli had the same color in half of the trials (singleton absent), whereas one of the four stimuli had a different color (singleton present) in the other half of the trials. The singleton was never the search target; thus, it functioned as a distractor. The color of the target stimulus was fixed (Experiments 1 and 3) or random (Experiment 2); the color of the distractor (singleton) was also fixed (Experiments 1 and 2) or random (Experiment 3). Notably, the task was just to identify the location of a dot inside the target (diamond), and the assigned color was irrelevant to the task. We examined whether or not the singleton-presence benefit can be obtained even when the target or distractor color was changed on a trial-by-trial basis. In the probe trials (bottom panel), the task was to detect a probe-target letter (A or B). In Experiment 1, in half of the trials, the target color (e.g., green) in the visual search was presented, while in the other half, the distractor color (e.g., red: a singleton color in the visual search) was presented. The probe-target letter appeared in a critical color (either of the target or distractor colors in search trials) or in a neutral color (a color that had not been presented in search trials). The probe presentation in the target or distractor feature that was presented in the search trials enabled the assessment of the target enhancement and distractor suppression separately. In Experiments 2 and 3, either the distractor or target color appeared in half of the probe trials, respectively, while all items were presented in neutral colors in the other half of the trials. This process enabled the calculation of the distractor suppression and target enhancement effects in Experiments 2 and 3, respectively.

- Reaction time is usually highly skewed distributed. It seems to me that the authors performed stats on the raw RT values. I suggest trying to perform stats on log-transformed RT. That should better fit the intentions of t-tests and ANOVAs.

Thank you for the suggestion. We used the same analysis using log-transformed RT data and obtained the same results. We added these analyses in Supplementary Analysis.

 Log-transformed RT was used for the analysis.

**Visual Search**

*Experiment 1*

 RTs were faster for singleton-present trials than in singleton-absent trials ( *t* (45) = 2.13, *p* = .039, *d* = 0.31).

*Experiment 2*

 No RT differences were observed between singleton-present and -absent trials ( *t* (47) = 0.37, *p* = .716, *d* = 0.05).

*Experiment 3*

 RTs were faster for singleton-present trials than in singleton-absent trials ( *t* (46) = 5.24, *p* < .001, *d* = 0.76).

**Probe Task**

*Experiment 1*

 No significant main effect of critical color and probe-target location on RT ( *F* (1, 45) = 1.23, *p *= .274, $\eta_{p}^{2}$ = .03; *F* (1, 45) = 0.74, *p *= .391, $\eta_{p}^{2}$ = .02) nor any significant interaction ( *F* (1, 45) = 2.20, *p *= .144, $\eta_{p}^{2}$ = .05) was found.

*Experiment 2*

 No difference was observed in the probe location for the suppression effect ( *t *(47) = 1.07, *p* = .290, *d* = 0.16).

*Experiment 3*

 No difference was observed in the probe location for the enhancement effect ( *t *(46) = 0.42, *p* = .674, *d* = 0.06).

- It remains unclear to me the rationales behind Experiments 2 and 3. The authors mentioned that “We hypothesized that if two attentional guidance elements, enhancement and suppression, competed for common processing resources, the magnitude of the effect in Experiment 1 would be smaller than those in Experiments 2 and 3 because Experiment 1 required both of these attentional controls.” This argument is unclear to me. If either the target or distractor color is randomly selected, the advantage of color expectation should be diminished and that would lead to a smaller enhancement or suppression effect. Can the authors elaborate on the rationales and the predictions?

Although we hypothesized that the target enhancement and distractor suppression will become larger in Experiments 2 and 3, a possibility indeed exists that these effects will become relatively smaller due to the smaller advantage of color expectation, as pointed out by the reviewer. In fact, we did not find larger target enhancement and distractor suppression in Experiments 2 and 3, which is partially due to the weaker color expectation effect. We included this aspect in the discussion.

 Although we hypothesized that the target enhancement and distractor suppression would increase in Experiments 2 and 3, a possibility exists that these effects would relatively decrease due to the smaller advantage of color expectation. The reason is that the target and distractor colors were fixed in Experiment 1, whereas the target or distractor color was randomized in Experiments 2 and 3. In fact, we were unable to identify large target enhancement and distractor suppression effects in Experiments 2 and 3, which is partially due to the weaker color expectation effect.

- “This singleton-presence benefit fits with earlier findings [8,10], allowing us to test target enhancement and distractor suppression for probe trials.” Here the numbered-style references should be replaced by inline-style references.

Thank you for pointing out this aspect. We replaced the references as follows:

 This singleton-presence benefit fits with earlier findings (Chang & Egeth, 2019; Gaspelin et al., 2015), allowing us to test target enhancement and distractor suppression for probe trials.

- The figure captions should be improved. For example, in Fig. 2, does the black horizontal line in the box plot represent the mean or median of the group? What are the definitions of the upper and lower edges of the boxes and of the range of the whiskers?

Thank you for the suggestion. We added the required information in the caption. For example, the caption for Figure 2 is presented as follows:

**Figure 2.** The mean reaction times (RTs) in the visual search task as a function of the singleton condition. Each dot represents the mean RT per participant. The RT was shorter in singleton-present trials than in singleton-absent trials for Experiments 1 and 3. The box represents the interquartile range, which contains 50% of the values (median with midline), and the bottom and top edges of the whiskers represent the 1.5 × interquartile range. Figures were drawn using the “raincloudplots” package in R (Allen et al., 2021).

# Peer review: Reviewer response for version 1

**DOI:** 10.5256/f1000research.81418.r140348

**Published:** 2022-06-27

**Authors:** Xiangyong Yuan

**Affiliations:** 1 State Key Laboratory of Brain and Cognitive Science, CAS Center for Excellence in Brain Science and Intelligence Technology, Institute of Psychology, Chinese Academy of Sciences, Beijing, China

The submitted paper re-examined an interesting issue whether target enhancement and distractor suppression in visual search are performed in parallel. The introduction was a thorough one, having reviewed several relevant studies, it raised a clear standpoint about why these two processes may not be weighted in an equal manner. Although failed to fully replicate a previous one, they observed a novel negative correlation between target enhancement and distractor suppression, and found that expecting only one target color compared with only one distractor color can benefit search. As the authors explained, online experiments may allow more personal strategies in search thus more individual variances than lab experiments. Overall, I agree with most of the authors’ opinion about the relationship between target and distractor suppression. Besides, I fell the experiments are well-conducted and the manuscript is well-written, and would be very glad to see it published after my minor concerns below are addressed:

1. The authors noticed that after few practice trials observers may not find a stable search strategies therefore have not shown simultaneous target enhancement and distractor suppression. Suppose the search strategies were stable for a particular observer only after enough practice trials, if the first few trials, or the first block in the formal experiment was abandoned, will these two processes be found like Chang and Egeth (2019)? Or alternatively, the author may split the data into first and second halves, examining how the search strategies change over time. This may also help to reveal the individual differences if they indeed have different strategies.
2. The mean accuracies of probe task in Chang and Egeth (2019) were above 90%. In contrast, the mean accuracies were below 80% in exp. 1–3. This may suggest that the participants in the current online experiments did not fully focus on the task, compared with in the lab. Is it possible that target enhancement and distractor suppression can only work in parallel with sufficient attention resources? The authors may select and analyze the data from those participants with higher accuracies that match those in Chang and Egeth (2019), and check if this is true. This possibility may be mentioned in the discussion.
3. In discussion, a conceptual suppression of the singleton has been proposed as a possible explanation of exp. 3. But it seems the search benefit in exp. 3 can be simply explained by a figure-background segregation. If all the targets share the same color across trials while the distractor is always distinguishable from the targets, the observers could learn to only search for the targets and simultaneously ignore the distractors as an irrelevant background. I am not sure whether we actually need to suppress the singleton on a conceptual (or semantic) level of saliency. The distractors can be easily excluded from the search pool due to their first order saliency.
4. In Page 4, the authors wrote: “Accordingly, we calculated the magnitude of the enhancement and suppression for each participant and compared the effect across experiments. We hypothesized that if two attentional guidance elements, enhancement and suppression, competed for common processing resources, the magnitude of the effect in Experiment 1 would be smaller than those in Experiments 2 and 3 because Experiment 1 required both of these attentional controls.” But I didn’t find this comparison in the result section.

Is the work clearly and accurately presented and does it cite the current literature?

Yes

If applicable, is the statistical analysis and its interpretation appropriate?

Yes

Are all the source data underlying the results available to ensure full reproducibility?

Yes

Is the study design appropriate and is the work technically sound?

Yes

Are the conclusions drawn adequately supported by the results?

Yes

Are sufficient details of methods and analysis provided to allow replication by others?

Yes

Reviewer Expertise:

multisensory integration, selective attention

I confirm that I have read this submission and believe that I have an appropriate level of expertise to confirm that it is of an acceptable scientific standard.

# Response

**Published:** 2022-09-16

**Authors:** Tomoya Kawashima

- The authors noticed that after few practice trials observers may not find a stable search strategies therefore have not shown simultaneous target enhancement and distractor suppression. Suppose the search strategies were stable for a particular observer only after enough practice trials, if the first few trials, or the first block in the formal experiment was abandoned, will these two processes be found like Chang and Egeth (2019)? Or alternatively, the author may split the data into first and second halves, examining how the search strategies change over time. This may also help to reveal the individual differences if they indeed have different strategies.

Thank you for the suggestion. We divided the trials and used the second half for analysis. The results illustrated that no distractor suppression was observed, although the target enhancement effect was significant. In a critical sense, a negative correlation once again exists between enhancement and suppression effects, which were observed for all data (Figure 5). Therefore, we deem that these data support our idea that attentional guidance is dependent on the search strategy of the participant. We added the following text:

 Chang and Egeth (2019) gave 32 probe and 48 search practice trials; to save time, we gave 10 probe and search practice trials. These differences in the exposure to target and distractor prior to the task might have led to the weak enhancement and suppression in the current study. To test this assumption, we divided trials into first and second halves and used the second half of the trials for analysis. The visual search performance pointed to the singleton-benefit effect ( *n* = 40, −7.2 ms [95% CI: −12.68 to −1.81]; *t* (39) = 2.70, *p* = .010, *d* = 0.43). For RTs in the probe task, the study observed a significant interaction ( *F* (1, 39) = 4.68, *p *= .037, $\eta_{p}^{2}$ = .11), which reflected a significant target enhancement effect ( *F* (1, 39) = 4.47, *p *= .041, $\eta_{p}^{2}$ = .10) but not a distractor suppression effect ( *F* (1, 39) = 1.07, *p *= .307, $\eta_{p}^{2}$  = .03). Importantly, the study also observed a significant negative correlation between enhancement and suppression effects ( *r* = −.34, 95% CI [−.59, −.03], *p* = .031). Thus, the study noted no clear distractor suppression effect even in the second half of the trials, where the participants were sufficiently exposed to the target and the distractor. Instead, the data support our idea that attentional guidance is dependent on the search strategy of the participants.

- The mean accuracies of probe task in Chang and Egeth (2019) were above 90%. In contrast, the mean accuracies were below 80% in exp. 1–3. This may suggest that the participants in the current online experiments did not fully focus on the task, compared with in the lab. Is it possible that target enhancement and distractor suppression can only work in parallel with sufficient attention resources? The authors may select and analyze the data from those participants with higher accuracies that match those in Chang and Egeth (2019), and check if this is true. This possibility may be mentioned in the discussion.

Thank you for the suggestion. In the current experiment, only a few particpants exhibited accuracies higher than 90%. We selected participants with relatively high accuracy rates (above 70%) in the probe task and assessed their RTs but found no target enhancement or distractor suppression effects. Therefore, high accuracy rates in the probe task do not seemingly guarantee the enhancement or suppression effect. We added the following text:

 Furthermore, the study observed that the mean accuracy of the probe task in Experiments 1–3 was less than 80%, whereas Chang and Egeth (2019) reported more than 90%. This difference may suggest that the participants in the online experiments only partially focused on the task compared with experiments conducted in laboratory settings. The current study investigated whether or not target enhancement and distractor suppression can work in parallel with sufficient attentional focus on the task in an exploratory manner. We selected participants with relatively higher accuracy (above 70%) on each condition of the probe task and re-analyzed their reaction times (see Figure 4 for the distribution of accuracy data). In Experiment 1, the study selected 19 out of 46 participants. They displayed no target enhancement effect (13.4 ms [95% CI: −16.2 to 43.0]; *t *(18) = 0.95, *p* = .353, *d* = 0.22) or distractor suppression effect (18.6 ms [95% CI: −13.7 to 50.9]; *t* (18) = 1.21, *p* = .243, *d* = 0.28). In Experiment 2, the study selected 28 out of 48 participants, which exhibited no distractor suppression (12.3 ms [95% CI: −24.7 to 25.7]; *t* (27) = 0.04, *p* = .969, *d* = 0.01). In Experiment 3, 27 out of 47 participants produced no target enhancement effect (12.2 ms [95% CI, −16.1 to 34.3]; *t* (26) = 0.74, *p* = .464, *d* = 0.14). Thus, even the participants who would have focused their attention on the task presented no target enhancement or distractor suppression. As such, higher accuracy in the probe task seemingly does not guarantee the enhancement or suppression effect.

- In discussion, a conceptual suppression of the singleton has been proposed as a possible explanation of exp. 3. But it seems the search benefit in exp. 3 can be simply explained by a figure-background segregation. If all the targets share the same color across trials while the distractor is always distinguishable from the targets, the observers could learn to only search for the targets and simultaneously ignore the distractors as an irrelevant background. I am not sure whether we actually need to suppress the singleton on a conceptual (or semantic) level of saliency. The distractors can be easily excluded from the search pool due to their first order saliency.

Thank you for the suggestion. We added the following statement in the discussion:

 Another concept for the mechanisms underlying the singleton-presence benefit is figure-background segregation. In Experiment 3, where the target color was fixed, and the distractor color was varied across trials, the participants learned to search for the target color while segregating the distractor colors as background, which led to the singleton-presence benefit. Thus, future studies are required to elucidate the mechanisms underlying the singleton-presence benefit.

- In Page 4, the authors wrote: “Accordingly, we calculated the magnitude of the enhancement and suppression for each participant and compared the effect across experiments. We hypothesized that if two attentional guidance elements, enhancement and suppression, competed for common processing resources, the magnitude of the effect in Experiment 1 would be smaller than those in Experiments 2 and 3 because Experiment 1 required both of these attentional controls.” But I didn’t find this comparison in the result section.

Thank you for the comment. We have planned but did not conduct this analysis, because we did not observe significant enhancement and suppression effects for all experiments. As suggested, we conducted this analysis with the following results. We added the following statement:

 Although the study observed no target enhancement or distractor suppression, we compared these effects across experiments. For the target enhancement effect, no significant difference was observed between Experiments 1 and 3 (Experiment 1: −1.7 ms; Experiment 3: −8.1 ms; *t* (91) = 0.49, *p* = .624, *d* = 0.10). Similarly, no significant difference was observed between Experiments 1 and 2 (Experiment 1: −13.5 ms; Experiment 2: 8.5 ms; *t* (92) = 1.68, *p* = .097, *d* = 0.35) for the distractor effect.

# Peer review: Reviewer response for version 1

**DOI:** 10.5256/f1000research.81418.r138946

**Published:** 2022-06-09

**Authors:** Li Jingling

**Affiliations:** 1 China Medical University, Taichung, Taiwan

This study tried to replicate Chang and Egeth (2019) and extended their conditions to a randomized target color or distractor color in separate blocks. Their goal was to test whether attentional setting on the task demand and distractor suppression are related or separate processing. They replicated Chang and Egeth (2019) in that presenting a distractor actually facilitated the speed of target discrimination, suggesting there was target enhancement and distractor suppression occurred simultaneously. Such effect increased when the target color was fixed and distractor color varied trial-by-trial, suggesting a varied distractor induced a stronger suppression. Nevertheless, when target color varied trial-by-trial and distractor color was fixed across trials, there was no distractor suppression. Also, the interleaved probe task with the search trials did not reveal any significant effect. The author found a negative correlation between target enhancement and probe suppression in the probe task in experiment 1, suggesting that those who enhance the target more tend to suppress distractors less. The authors also provided a meta-analysis of their own data and that of Chang and Egeth (2019) and obtained a significant correlation effect. The author, therefore, concluded that the two operations, target enhancement and distractor suppression, are related. 

 This study is well-conducted in method, the data are well processed, and the conclusion is clear and potentially contributes to academic literature. I suggest accepting this article with minor revisions:

 1. I have difficulty understanding why non-significant probe data can generate a significant correlation in experiment 1, and why such corresponding analysis cannot apply to experiments 2 and 3. Does that mean a traditionally assumed distractor capture also occurred? Can probe data in experiments 2 and 3 also be considered separated into "target" or "distractor" colors even if they are varied? 

 2. I like your strategy account, which is consistent with the definition of attention in resource control. Could it be possible that online data collection essentially probes a different group of participants compared to that recruited by Chang and Egeth (2019)? For instance, those who come to the lab to complete the task might be mainly graduate students while those who complete an online experiment are more close to the heterogeneous population? The authors may add some discussion on this point.  

 3. I noticed that the accuracy of experiment 2 was a bit lower than that in experiments 1 and 3, is there any significance? Randomizing target color across trials may make the task more difficult, even if the target was not defined by color. This can also be a piece of evidence on the importance of strategy induced by different task settings.

Is the work clearly and accurately presented and does it cite the current literature?

Yes

If applicable, is the statistical analysis and its interpretation appropriate?

Yes

Are all the source data underlying the results available to ensure full reproducibility?

Yes

Is the study design appropriate and is the work technically sound?

Yes

Are the conclusions drawn adequately supported by the results?

Partly

Are sufficient details of methods and analysis provided to allow replication by others?

Yes

Reviewer Expertise:

cognitive psychology, human attention, cognitive control

I confirm that I have read this submission and believe that I have an appropriate level of expertise to confirm that it is of an acceptable scientific standard.

# Response

**Published:** 2022-09-16

**Authors:** Tomoya Kawashima

- I have difficulty understanding why non-significant probe data can generate a significant correlation in experiment 1, and why such corresponding analysis cannot apply to experiments 2 and 3. Does that mean a traditionally assumed distractor capture also occurred? Can probe data in experiments 2 and 3 also be considered separated into "target" or "distractor" colors even if they are varied?

Thank you for the comment. Our interpretation of the observed negative correlation between target enhancement and distractor suppression is that “whether attentional enhancement or distractor suppression works depends on the observer’s choice of search strategy”. In other words, individuals will adopt a strategy to focus their attention on the target dimension, whereas others will adopt a different strategy to focus on the distractor dimension. This variability in strategy across participants may be “one reason why no attentional enhancement or distractor suppression was observed in group-level analysis.”

 We cannot apply the same analysis to Experiments 2 and 3 for distractor suppression and target enhancement, respectively, because different participants in each experiment were different. Therefore, we cannot consider the correlation between Experiments 2 and 3 (please see Figure 5).

 The probe data in Experiments 2 and 3 were obtained with a fixed distractor or target color, respectively. Therefore, we can consider the data from these experiments as producing the distractor suppression and target enhancement effects.

- I like your strategy account, which is consistent with the definition of attention in resource control. Could it be possible that online data collection essentially probes a different group of participants compared to that recruited by Chang and Egeth (2019)? For instance, those who come to the lab to complete the task might be mainly graduate students while those who complete an online experiment are more close to the heterogeneous population? The authors may add some discussion on this point. 

Thank you for the suggestion. We have added the following sentence:

 In addition, the online experimental settings enable the collection of various populations compared with laboratory experiments. Hence, the difference in data from online and laboratory experiments should be interpreted with caution.

  

- I noticed that the accuracy of experiment 2 was a bit lower than that in experiments 1 and 3, is there any significance? Randomizing target color across trials may make the task more difficult, even if the target was not defined by color. This can also be a piece of evidence on the importance of strategy induced by different task settings.

Thank you for the suggestion. We performed the additional analysis to compare the accuracy of the experiments and found no significant difference. Therefore, we concluded that randomizing the target or distractor color did not change the overall difficulty of the probe task. We added the following sentences:

 We further compared the accuracy of the experiments to verify whether the overall task difficulty differed among the experiments. In the target-color present condition (Figure 4B and D), two-way ANOVA with between-subject factor (experiment) and within-subject factor (probe condition) demonstrated the lack of the main effect of the experiment or probe condition ( *F* (1, 91) = 0.24, *p *= .629, $\eta_{p}^{2}$ = .00; *F* (1, 91) = 0.16, *p *= .695, $\eta_{p}^{2}$ = .00) or an interaction effect ( *F* (1, 91) = 0.43, *p *= .512, $\eta_{p}^{2}$ = .00). The same analysis was performed for the distractor-color present condition (Figure 4A and C), which illustrated the absence of the main effect of the experiment or probe condition ( *F* (1, 92) = 0.005, *p *= .944, $\eta_{p}^{2}$ = .00; *F* (1, 92) = 0.22, *p *= .639, $\eta_{p}^{2}$ = .00) or an interaction effect ( *F* (1, 92) = 0.06, *p *= .809, $\eta_{p}^{2}$  = .00). Thus, randomizing the target or distractor color did not change the overall difficulty of the probe task.
